# Sensory Characterization and Acceptability of a New Lulo (*Solanum quitoense* Lam.) Powder-Based Soluble Beverage Using Rapid Evaluation Techniques with Consumers

**DOI:** 10.3390/foods11193129

**Published:** 2022-10-08

**Authors:** María Remedios Marín-Arroyo, Sofía Marcela González-Bonilla

**Affiliations:** Institute for Sustainability & Food Chain Innovation (ISFOOD), Universidad Pública de Navarra, 31006 Pamplona, Spain

**Keywords:** CATA, check-all-that-apply, consumer acceptance, penalty analysis, powdered beverage, tropical fruits

## Abstract

Recently, the interest in tropical fruits has increased widely even beyond their production areas, but the perishable nature of these fruits makes their marketing difficult. However, due to its special sensory characteristics and nutritional value, lulo (*Solanum quitoense* Lam.) is a good candidate for product development to meet this ever-growing demand. Therefore, a lulo-powder-based soluble beverage was prepared according to previously established formulations. Thus, the aim of the present research was to obtain the sensory characterization, study consumers’ overall acceptability, and identify drivers of liking for the new beverage. Eight samples were prepared with lulo juice or pulp + stevia, or a sweetener blend (erythritol + xylitol + stevia). Maltodextrin or inulin, as a drying aid, was added to freeze-dry the samples. The freeze-dried samples were rehydrated for consumption. The sensory characterization of the new beverage was carried out by using CATA questions with consumers (*n* = 69). The most influential attributes that affected acceptability were identified by using ideal product characterization and hedonic scores of the samples. The beverage formulations with stevia alone had the lowest acceptability. Most sensory differences among samples were found between the visual attributes. The attributes “clean”, “homogeneous”, “fruity” and “citrus” odor, “just-right acidity”, “just-right sweetness”, and “fresh” were necessary to increase global acceptance in the juice-only beverages (Js), whereas “cloudy”, “off-odor”, and “very acidic” negatively impacted acceptance. For products with pulp (Ps), “citrus” and “tropical fruit” odors, “just-right acidity”, “just-right sweetness”, and “fresh” attributes were needed to increase acceptance, while “cloudy” and “chemical/artificial” flavors negatively impacted acceptance. The lulo-powder-based soluble beverage was accepted by consumers; however, there is still potential for the sensory-quality improvement of this product.

## 1. Introduction

Lulo (*Solanum quitoense* Lam.) is a tropical fruit that is commonly cultivated in Central and South America [1]. However, its main markets outside of its production areas are the United States and Europe [1,2]. In recent years, interest in tropical fruits has increased in non-producing countries. Products such as tropical fruit juices are gaining market share in Europe, particularly those with outstanding nutritional values and proven health benefits [3]. Lulo fruit has generated interest in these new markets due to its sensory [4,5] and biofunctional properties [6]. Furthermore, import demand for this fruit has evolved with migration, as consumers tend to maintain their food preferences [7]. The lulo fruit is rarely consumed fresh, mainly because of its acidity. It is commonly used to produce flavored drinks, preserves, and desserts. Fresh juice is also processed into frozen concentrates and can be fermented to produce wine [8]. Lulo is most commonly consumed as nectar [9,10]. Fruit nectar is prepared by mixing fruit pulp with water and sugar. Lulo nectar is usually prepared at 13 to 15 °Brix, of which about 5 °Brix is provided by the fruit [11]. Dilution with water can be highly variable: water:pulp at 1:1 to 3:1 [12] and water:pulp at 2:1 [11].

One of the problems associated with lulo, as well as all fruits, is that it is highly perishable, thus making its distribution difficult. The transformation of the fruit into low-perishable, storable, and marketable products adds economic value to the raw material, reduces waste, and facilitates better use of the fruit [13,14].

Research on the development of new products based on lulo has increased in recent years. Products such as jams [15], carbonated beverages [16], and alcoholic beverages [17] have been developed. Its use as a possible ingredient has also been investigated; the fatty acid content of its seeds has shown potential as an oleaginous raw material (fatty oils from seeds) [18], along with the derivation of other possible ingredients, such as bagasse powder [6], peel powder [19], and juice powder [4,6,8,10,20].

The production of a drink obtained by rehydrating powdered fruit, without added sugars, would be a suitable new alternative in product development. Dehydration would provide a product that is easy to preserve and transport, with better quality properties compared to the flavored powders that are currently available on the market.

Among food-processing operations, drying is one of the earliest and remains one of the most common food-preservation techniques. Drying has numerous benefits, including increased product stability during storage, reduced packaging requirements, and less mass to transport [21]. Several factors are important to consider when choosing a drying process. The process must be cost-effective. It is also important that the drying process reduces fresh-material degradation, along with allowing for the retention of as much of the sensory and nutritional properties of the original material as possible [21]. Forero et al. [4,20] compared distinct lulo drying methods (hot-air-drying, ultrasound-assisted hot-air-drying, freeze-drying, and spray-drying); they concluded that, considering the thermal stability and potential biofunctional ingredients of the products obtained, freeze-drying and spray-drying were the most suitable methods for the development of healthy products [20]. However, freeze-drying was the best method for obtaining lulo powder, due to its high drying yield, low water activity, and excellent retention of the active volatile aromas that resemble fresh fruit [4]. In fruit juices, the presence of organic acids and other low-molecular-weight compounds, such as sugars, hinders the conversion of juice to powder due to factors such as the low glass transition temperature and high hygroscopicity, which can reduce yield and cause operational problems. The use of drying aids can help to reduce these problems [22,23,24]. Maltodextrin is one of the most common drying aids used, as it is cost-effective, flavorless, and odorless [25]. Other options include inulin, which has prebiotic properties; is a good source of fiber; and is a good stabilizer, cryoprotectant, and encapsulating agent [26]. Inulin has been used as a drying aid in different juices, such as orange [27], cranberry [22], and passion fruit [23].

Excessive sugar consumption is a driving factor in epidemics such as obesity and non-communicable diseases [28]. Therefore, the use of sweeteners is a good option to reduce sugar intake [29]. In the selection of sweeteners, it is important to consider their physicochemical characteristics, caloric contribution, and sensory profile. Among the viable options, polyalcohols provide volume, texture, and low-calorie content [30] and can mask residual flavors [30]. Stevia, a safe natural sweetener, is a zero-calorie sweetener that is 470 times sweeter than sucrose [29].

The final receivers of agrifood products are consumers who demand the maintenance of natural fresh characteristics in processed products. Therefore, in the development of new food products, to address the needs of consumers, it is essential to obtain information about their perception of the sensory characteristics, their overall acceptance (liking/disliking), and the attributes of the product that they appreciate the most or dislike. For this purpose, new rapid sensory-analysis methods, in particular, the check-all-that-apply (CATA) method, have been proven to be very useful, reliable tools [31].

Since its introduction in 2007 [32], CATA has proven to have a discriminatory power that is comparable to other methods, such as intensity ratings or napping [33]. Its utility as a profiling method when used with consumers [34,35,36] has also been demonstrated. In addition, when combined with ideal product evaluation and hedonic evaluation of samples, the CATA method can provide useful information related to product characteristics that increase or decrease acceptance.

The CATA method consists of a multiple-choice question in which judges are presented with a list of words or phrases and asked to select all the options that they consider to be present in or applicable to the product under evaluation [37]. To obtain information about consumers’ drivers of liking, following the CATA questionnaire, consumers are asked to select the terms that they consider appropriate to describe their ideal product. The data are analyzed by counting the number of consumers who did not select the same attributes as they did for their ideal product, along with the associated reduction in the overall acceptance mean drop [38].

In the present research, CATA questions combined with ideal product (IP) and hedonic scores were used to describe the sensory characteristics and identify consumer acceptability and the drivers of liking of a new lulo (*Solanum quitoense* Lam.)-powder-based soluble beverage.

## 2. Materials and Methods

### 2.1. Raw Material

Lulo de Castilla (*Solanum quitoense* Lam.) from Colombia (Productos Goya Nativo, S.L. Colombia) packed as frozen whole fruit in 387 g bags; citric acid, maltodextrin, and inulin (Guthaus, molecular cuisine, Navarra, Spain); ascorbic acid (Nutrivita, Toulouse, France); erythritol, xylitol, and stevia (NKD Living, London, UK); and low-mineralization natural mineral water (Fuencisla, Valencia, Spain) were purchased from local suppliers and retailers.

The ripening stage of the lulo fruit was established according to the classification system of the Colombian technical standard NTC 5093 (Instituto Colombiano de Normas Técnicas y Certificación, 2002). Among them, 16% of the fruit was at maturity stage 3 (orange fruit with green hints in the center of the fruit), 41% at maturity stage 4 (orange fruit with few green hints), and 43% at maturity stage 5 (orange fruit).

### 2.2. Preparation of Formulations before Dehydration

#### 2.2.1. Beverage Formulation

To establish the formulation of the new drink, the ideal sweetness, acidity, and dilution of the lulo beverages made with juice only or with pulp were previously investigated [39] by using an acceptance test and just-about-right (JAR) scales. Two factors were considered in the design: factor W (water/fruit proportion), ranging from 0.67 to 3.00, and factor S (sucrose/(fruit + water)), ranging from 0.06 to 0.14 (sucrose concentration ranging from 6 to 14%). The samples with water/juice only 0.95 (factor W = 0.95) and 14% sucrose (factor S = 0.14), and water/pulp 1.96 (factor W = 1.96) with 14% sucrose (factor S = 0.14) presented the highest overall acceptance and were closest to the ideal.

Based on these results, eight beverage samples were prepared: four made with juice only (Js) and four made with pulp (juice + mesocarp) (Ps). The new samples were formulated as follows: factor W = 0.95 for Js samples and factor W = 1.96 for Ps samples were used; sucrose was replaced with the sweeteners stevia (S) or a mixture of erythritol, xylitol, and stevia (X) in the quantities necessary to ensure a sweetening effect equivalent to that of factor S = 0.14. This adjustment was accomplished by taking into account that the sweetening power of xylitol is the same as that of sucrose, sucrose is 1.3 times sweeter than erythritol, and stevia is 470 times sweeter than sucrose. Maltodextrin (M) or inulin (I) was used as a drying aid in a concentration equivalent to 10% of the fruit [8]. Citric and ascorbic acids were used to reduce color changes due to enzymatic browning in the pulp [40]. The detailed formulations are listed in Table 1.

#### 2.2.2. Pulp and Juice Extraction

Prior to juice or pulp extraction, the whole fruits were washed and disinfected by immersion in a 100 ppm sodium hypochlorite solution for 5 min, after which they were dried with a paper towel.

To obtain the juice, the fruit was cut in half with a knife, after which the juice, seeds, and placenta were extracted manually. The juice was separated by using a 710 µm sieve (Retsch, Haan, Germany).

To obtain the pulp, the exocarp was separated manually; the fruit was cut in two with a knife; and the mesocarp was separated from the juice, seeds, and placenta. The juice was then separated by using the same procedure as mentioned above with the sieve. The mesocarp was immersed in a solution of type II water containing 0.3% citric acid + 1.7% ascorbic acid, and then it was cooled to 2.1 ± 1.3 °C with an ice cover for 1 h. The mesocarp was drained, and then the previously obtained juice was added. Depending on the amount of fruit (juice + mesocarp), 0.3% citric acid and 1.5% ascorbic acid were added. The mixture was ground in a Thermomix (TM5, Vorwerk, Germany), at speed 10, for 45 s; to obtain a homogeneous mixture, it was filtered with a 710 µm sieve (Retsch, Haan, Germany).

#### 2.2.3. Additive Incorporation

The corresponding drying aid (maltodextrin or inulin) and sweetener (stevia only) were added to half of the juice or pulp. The additives were stirred to dissolution with a spoon until no lumps were observed. Subsequently, the remaining half of the juice or pulp was added and mixed again.

#### 2.2.4. Homogenization

The mixture was homogenized with an Ultra Turrax (Model T50 basic, IKA-WERKE, Staufen, Germany), at 8800 rpm, for 2 min. During homogenization, the vessel was kept refrigerated on a bed of ice.

### 2.3. Lulo-Powder Obtention

Dehydration was carried out by freeze-drying, using semi-industrial equipment (LYOBETA-25; Telstar Industrial, S.L. Barcelona, Spain). The freeze-drying conditions were stated in previous experiments carried out with the aim of obtaining a non-sticky, soluble dehydrated product with a moisture content below 7% (data not shown) and were as follows:

- Freezing phase: cooling to 5 °C for 30 min, then temperature reduction to −60 °C at 0.5 °C/min, and then the conditions were maintained at −60 °C for 12 h;
- Primary drying: temperature was increased to −40 °C at 0.2 °C/min, with vacuum pressure of 0.05 mbar, and then the conditions were maintained for 38 h;
- Secondary drying: temperature was raised to 25 °C at 0.2 °C/min, at maximum vacuum pressure, and then the conditions were maintained for 7 h.

The total drying process took approximately 68 h.

Freeze-dried samples were ground in a Thermomix (TM5, Vorwerk, Wuppertal, Germany) at speed 9 for 15 s and sieved with a 450 µm sieve (Retsch, Haan, Germany). Erythritol and xylitol powder (particles smaller than 450 µm) were added to the appropriate samples according to the formulation (Table 1).

### 2.4. Rehydration

The powdered lulo was rehydrated by adding low-mineralization natural water. The amount of water added was the sum of the water removed during freeze-drying plus the water corresponding to each sample according to its formulation.

### 2.5. Sensory Study

#### 2.5.1. Participants

Consumers (*n* = 69) were recruited based on their interest and availability to participate in the study from the consumer database of the Aenoltec research group of the Public University of Navarra, from university students and workers, and through snowball recruitment. The participants’ age distribution was 21–30 (42%), 31–40 (12%), 41–50 (17%), 51–60 (16%), and > 60 (13%). The female/male ratio (%) was 61/39. Among the participants, 29% were familiar with lulo, 59% consumed exotic or tropical fruits, and 85% consumed natural and different types of commercial juices. Cash incentives were not provided.

#### 2.5.2. Tasting Room Conditions

Sensory tests were conducted at the sensory-analysis laboratory of the Public University of Navarra in a standardized test room (ISO 8589:2007), at room temperature.

#### 2.5.3. Sample Presentation

All samples were tested by all the judges. Thirty milliliters of each sample was served in disposable glasses labeled with random 3-digit codes. Samples were presented in monadic sequence. The order followed a completed block design balanced for carry-over and position effects, using Williams’ Latin square design [41]. Participants were provided with water for rinsing between samples.

#### 2.5.4. Sensory Tests

Participants were first asked to score their acceptance (liking/disliking) by using a horizontal nine-point hedonic scale ranging from 1 = “extremely dislike” to 9 = “extremely like”.

Participants were then asked to select the attributes that best described the sample that they had evaluated, with no limit on the number of attributes that could be selected. The list of attributes included only terms related to sensory characteristics. A total of 50 terms (12 related to visual, 17 to odor, and 21 to flavor characteristics) were included in the CATA questions. The terms were selected based on previous works from the literature that evaluated fruit juices [42,43] and lulo [4]. The terms included in the CATA questionnaire and the abbreviations used to ease visualization of the graphics are listed in Table 2. The terms were separated into three sections (visual, odor, and flavor). To minimize the bias of the judges’ responses due to the position of the attributes in the checklist, each participant received the CATA questions with the terms in a different order [44]. The order in which the terms were included in the CATA questions was set by using Williams’ Latin square design [41].

Finally, participants were instructed to think about what characteristics this type of product should have in order to be considered their ideal product. From the list of descriptors provided, they were asked to pick those that best described their ideal product.

The data were collected by using the data-acquisition software Compusense Cloud 7.8.2 (Compusense Inc., Guelph, ON, Canada).

#### 2.5.5. Ethical and Security Aspects

In accordance with the points covered by the Ethics, Animal Experimentation, and Biosafety Committee of the Public University of Navarra, only those aspects related to the protection of personal data are applicable to the present study. Written consent was obtained from each participant prior to his/her participation in the study. In addition, as the sensory tests were carried out during the COVID-19 pandemic period, a protocol specifically developed for working in sensory-analysis facilities that had been validated by the Occupational Health Unit of the university was followed during the tests.

### 2.6. Statistical Analysis

A one-way analysis of variance, followed by a Fisher’s least-significant-differences (LSDs) test (*p* < 0.05), was performed on the hedonic evaluations to determine differences between samples.

A non-parametric Cochran’s Q test, together with a post hoc multiple-pairwise-comparisons Sheskin critical-difference test [45], was applied to the binary data obtained by the CATA method. These tests permitted the analysis of significant differences (*p* < 0.05) between samples for each attribute.

A contingency table was determined by counting the number of consumers that checked each term to describe each sample. A multiple correspondence analysis (MCA) [46] was performed on the contingency table, using χ^2^ distance, to explore the degree of relationship between juice samples and attributes. The attributes included in the MCA were those with a *p*-value below the 0.1 threshold.

A principal component analysis (PCA) [47] was applied to the correlation coefficients between attributes (tetrachoric correlation) and liking scores (biserial correlation) to obtain the sensory profile of the juice samples.

A penalty analysis of the CATA data, including the ideal product data, was applied to study the effect of each attribute on the judges’ mean hedonic scores [38] and obtain drivers of liking.

All statistical analyses were carried out by using XLSTAT software version 2020.2.3. (Addinsoft, Paris, France).

## 3. Results

### 3.1. Consumers’ Sensory Description of the Lulo-Powder-Based Soluble Beverage

The relevance of each term included in the CATA questionnaire to describe the characteristics of the lulo-beverage samples was determined by calculating the selection frequency. Supplementary Tables S1–S3 contain the frequencies (%) of selection of all terms for the products and the ideal product.

Cochran’s Q test was run independently for each attribute in the judges by products table. The Q test was followed by the Sheskin multiple comparisons test, which permitted the comparison of the distinct products. The results are listed in Table 3. The judges perceived differences in 10 out of 12 visual attributes, 3 out of 17 attributes related to odor, and 4 out of 21 flavor attributes.

Among the main results observed in Table 3, there is a clear distinction between two groups (Js and Ps); visually, Js samples and Ps samples differ in color. Js samples were more often described as green, while Ps samples were more often described as yellow. Sample JIS was perceived as cloudy a significantly higher number of times. Regarding flavor characteristics, Js samples were perceived as being very acidic more times than the Ps samples (Js samples significantly differ (*p* < 0.05) from PIS, PMX, and PIX). The attributes related to aroma from the JIS sample were more often described as aromatic, thus differentiating JIS from the PIX sample, which was more often perceived as being not very aromatic. The judges perceived chemical flavor more often in the samples containing stevia, and to an even greater extent in the samples with pulp.

The ideal product was described by the judges as having a homogeneous appearance (72.5%), very intense color (56.5%), clean (53.6%), bright (44.9%), yellow color (43.5%), tropical fruit (63.8%), fruity (53.6%) and citrus odor (50.7%), and very aromatic (43.5%), with just-right acidity (66.7%), sweetness (53.6%), and fresh flavor (56.5%). In the following formulations, a high percentage of judges found desirable attributes they had previously described for the ideal product: PIX (“yellow color” (68.1%), “fruity” odor (40.6%), and “just-right acidity” (40.6%)), JIX (“tropical fruit” odor (40.6%) and “just-right acidity” (46.4%)), PMX (“yellow color” (66.7%) and “tropical fruit” odor (46.4%)), PMS (“yellow color” (63.8%)), and PIS (“yellow color” (63.8%)).

For subsequent analyses, the following attributes that were considered non-significant (*p* > 0.10) were omitted: V–Homogeneous, O–Fruity, O–Floral, O–Chemical, O–Unripe, O–T.Fruit, O–Moldy, O–Sweet, O–Medicine, O–Off, O–Pineapple, O–Lemon, O–Orange, F–V.Sweet, F–N.Sweet, F–Bitter, F–Unripe, F–Rough, F–Dryness, F–Liquorous, F–Pineapple, and F–Orange.

**Figure 1:**
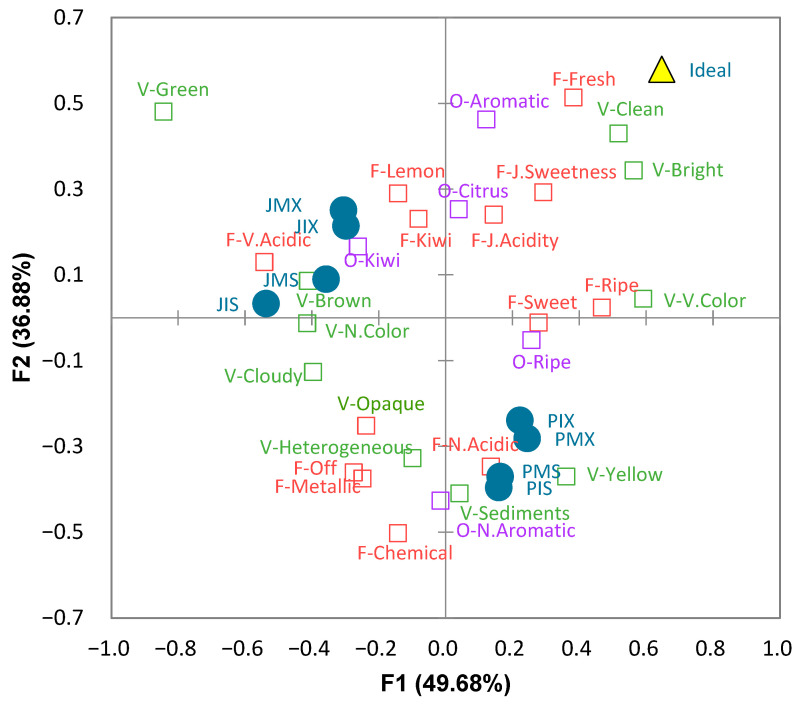
Representation of the lulo beverage samples (JMS, JIS, JMX, JIX, PMS, PIS, PMX, and PIX), attributes and ideal product in the first two dimensions of the multiple correspondence analysis (MCA) of the check-all-that-apply (CATA) counts. P = pulp, J = juice, M = maltodextrin, I = inulin, S = stevia, and X = erythritol + xylitol + stevia.

The similarities and differences between the samples, as well as their main characteristics, were distinguished with a multiple correspondence analysis (MCA) run on the contingency table. The results are shown in Figure 1. The two first dimensions explained 86.56% of the total variance.

Pulped and non-pulped products were located in different quadrants. Among each type of sample (Js and Ps), clusters were observed between samples with stevia (JMS and JIS; PMS and PIS) and samples with the sweetener mix (JMX and JIX; PIX and PMX); however, no association was observed between samples with stevia or the sweetener mix with specific attributes. Both pulped and non-pulped products were far from the ideal product that should be clean, bright, and fresh, according to the map of the analysis.

The correlation matrix including attributes and liking scores (data not shown) indicated that liking scores were positively, although weakly, correlated with “just-right acidity” (0.327) and “just-right sweetness” (0.354); they were negatively correlated with “off flavor” (—0.427) and “chemical/artificial” flavor (—0.426).

The principal component analysis (PCA) applied to the correlation coefficients confirmed that “clean”, “bright”, “just-right acidity”, “just-right sweetness”, and “fresh flavor” were associated with liking (Supplementary Figure S1).

To better visualize the characteristics of each group of samples, a new analysis was run for each sample type (Js and Ps). Table 4 contains the results of the Cochran’s Q test, followed by the Sheskin multiple comparisons test that was run for each type of sample.

Among the Js samples, JIS stood out for being associated with the visual attributes cloudy and opaque more often, and samples with stevia were associated with bitter, metallic, and chemical flavors, although the differences were not significant in some cases. Among the Ps samples, PMS was perceived as more homogenous than PIX; as in the Js samples, those with stevia were characterized by a higher frequency of mention for negative attributes, such as medicine odor, off-flavor, and chemical/artificial flavor.

**Figure 2:**
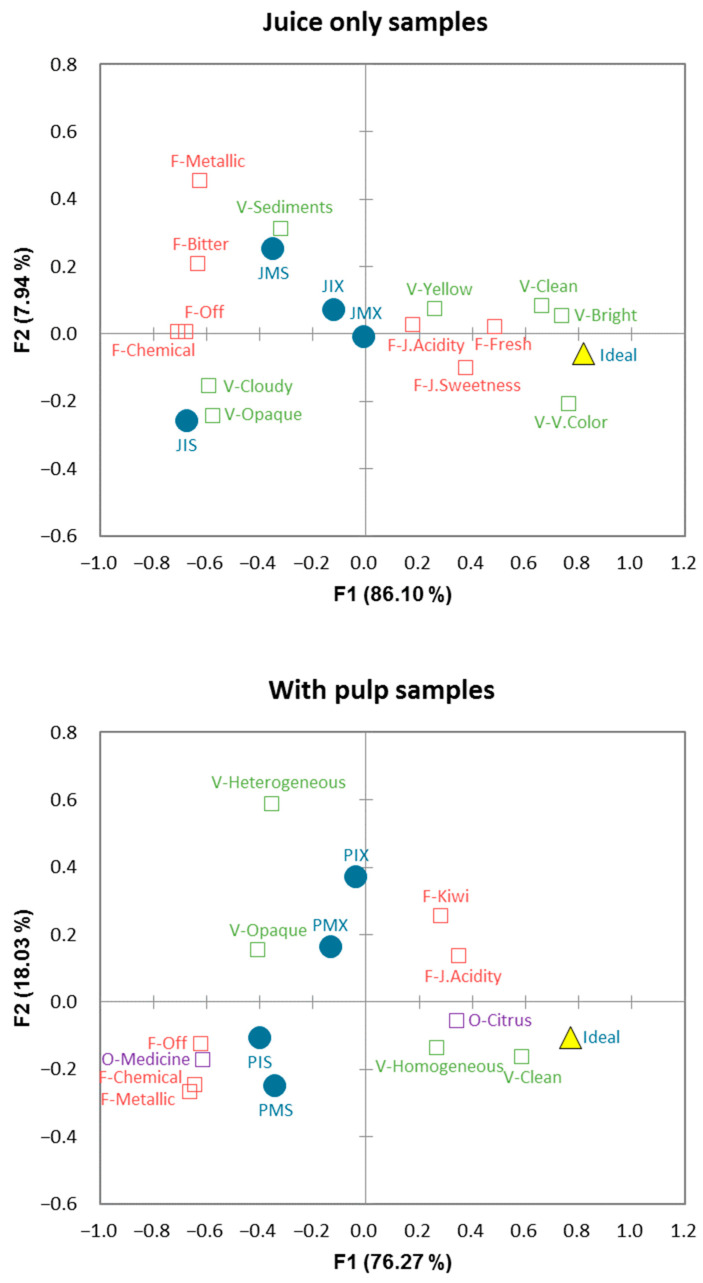
Representation of the lulo beverage samples (JMS, JIS, JMX, JIX, PMS, PIS, PMX, and PIX), attributes, and ideal product in the first two dimensions of the multiple correspondence analysis (MCA) of the check-all-that-apply (CATA) counts for the two groups of samples (juice only and with pulp). P = pulp, J = juice, M = maltodextrin, I = inulin, S = stevia, and X = erythritol + xylitol + stevia.

Figure 2 presents the results of the multiple correspondence analysis (MCA) run independently for each type of sample (Js and Ps). The two first dimensions explained 94.07% of the total variance of the Js samples and 94.30% of the Ps samples. JIS was mainly related to cloudy, opaque, chemical/artificial flavor, and off-flavor. JMS was located close to sediments, metallic, bitter, and off-flavor. Pulped samples with and without stevia were in different quadrants. Pulped samples with stevia were close to medicine odor and chemical, metallic, and off-flavors, whereas PMX was mainly related to opaque, and PIX was more related to heterogeneous. Both groups of samples were located far from the ideal. 

### 3.2. Consumers’ Overall Acceptability

The results of the hedonic evaluation are presented in Table 5. The average hedonic rating of the samples ranged from 5 = “neither like nor dislike” to 6 = “like slightly”. The samples with the highest scores were PMX, JMX, PIX, and JIX, corresponding to the products containing the sweetener blend (X), while the products with stevia (S) obtained the lowest scores.

### 3.3. Penalty Analysis to Identify Drivers of Liking

When judges are asked to describe both the samples and their ideal product, the penalty analysis can be used to determine the decrease in overall liking associated with a deviation from the ideal for each attribute [41]. The “must have” attributes (attributes that must be present in order not to cause a large decrease in liking) were identified by using the analysis of the attributes that were present in the ideal but missing in the real product (Supplementary Tables S4 and S6). A second analysis of the attributes missing in the ideal but present in the real product permitted the identification of the “must not have” (attributes that must not be present to avoid a drop in liking; see Supplementary Tables S5 and S7), “nice to have” (attributes that, if present in the real product, increase acceptability even if not selected for the ideal), and “indifferent” attributes (attributes of which presence or absence did not influence liking).

**Figure 3:**
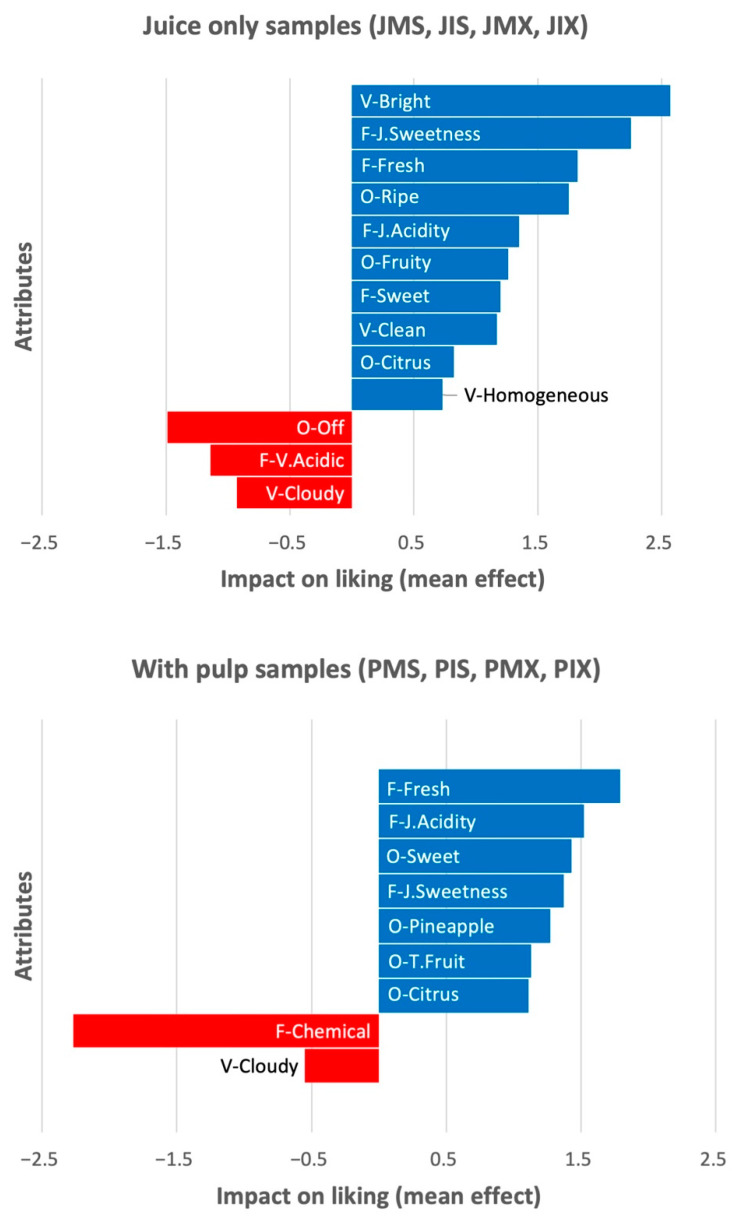
Penalty analysis. Attributes with a significant (*p* < 0.05) positive (blue color) or negative (red color) impact on the average liking scores. P = pulp, J = juice, M = maltodextrin, I = inulin, S = stevia, and X = erythritol + xylitol + stevia.

Given the differences in the characteristics of the Js and Ps samples, the penalty analysis was carried out independently for each type of sample. Figure 3 illustrates the attributes that positively or negatively impact the average liking scores.

The decision to include attributes in one of the three groups, necessary (“must have”), negative (“must not have”), and indifferent, was made by considering their impact on acceptability (Figure 3) and the percentage of consumers for whom there was an inconsistency (attribute selected in the ideal product and not in the real product or vice versa). The chosen threshold for population size was 20%. In the Js samples group, the attributes “clean”, “homogeneous”, “fruity” and “citrus” odor, “just-right acidity”, “just-right sweetness”, and “fresh” were necessary; the attributes “cloudy”, “off-odor”, and “very acidic” were negative; and “very intense color” and “tropical fruit” odor had no influence. For Ps samples, “citrus” and “tropical fruit” odor, “just-right acidity”, “just-right sweetness”, and “fresh flavor” were necessary; “cloudy” and “chemical” flavor were negative; and “clean”, “homogeneous”, “heterogeneous”, “very intense color”, and “fruity odor” had no influence.

## 4. Discussion

The products were statistically significantly different (*p* < 0.05) in regard to 17 of the 50 attributes presented; most of them were in the visual-attributes category (“cloudy”, “clean”, “opaque”, “green color”, “yellow color”, “heterogeneous appearance”, “sediments”, “very intense color”, and “bright”), three were from the odor-related attributes (“very aromatic”, “not very aromatic”, and “ripe fruit” odor), and four were flavor-related attributes (“sweet”, “very acidic”, “just-right acidity”, and “chemical/artificial” flavor). The products were clearly differentiated by color, as those made without pulp were mostly perceived as green, while those made with pulp were mostly perceived as yellow. Individually, the JIS sample differed from the rest due to being perceived more frequently as cloudy. Yellow-color perception in juices with pulp can be explained by the presence of the mesocarp part of the fruit; the mesocarp has a marked yellow color, which predominates in the products in which it is incorporated. Some studies [48] have reported that the incorporation of fruit mesocarp (passion fruit) in the formulation of products such as fresh pasta has a significant impact on color. Other studies on the sensory characterization of juice, such as one that carried out rehydration of freeze-dried and spray-dried grapefruit [49], also presented a clear difference in color between samples with and without pulp.

The ideal product described by more than 40% of the judges should have a homogeneous appearance; very intense color; be clean, bright, with yellow color; have a tropical fruit, fruity, and citrus odor; be very aromatic; have just-right acidity and just-right sweetness; and be fresh. The desirable attributes of the ideal product found by more than 40% of consumers were noted in the following samples: PIX (“yellow color”, “fruity” odor, and “just-right acidity”), JIX (“tropical fruit” odor and “just-right acidity”), PMX (“yellow color” and “tropical fruit” odor), PMS (“yellow color”), and PIS (“yellow color”). Samples elaborated with pulp were described by more than 63% of the consumers as “yellow”, while samples elaborated without pulp were described as “green”. More consumers found sediments in samples with pulp, while samples with juice only were most often described as “cloudy” and “opaque” in some cases (JIS). Selection frequencies for odor-related attributes were generally below 40%. Only the “tropical fruit” odor exceeded 40% in the JMX, JIX, and PMX samples, while the “fruity” odor and the descriptor “not very aromatic” exceeded 40% in the PIX sample. Among the flavor-related descriptors, those referring to acidity (“just-right acidity” and “very acidic”) were the most frequently selected to describe the samples. Products made without pulp were described as “very acidic” or “just-right acidity” with similar frequency in some cases (JMS and JMX).

The characteristics of the samples evaluated were far from the ideal product; they should be homogeneous, clean, bright, with very intense color, yellow, very aromatic, fruity, with tropical fruit and citrus odor, and fresh, with just-right acidity and just-right sweetness. The samples containing pulp were perceived as having a yellow color, having sediments, and as being not very aromatic. The samples without pulp were perceived as being green and cloudy. Although for a significant percentage of consumers, these samples had the right acidity; a large number of consumers perceived these samples as having very acidic flavor. Lulo is a fruit with a high content of organic acids, and citric acid represents more than 30% of the solids and 97% of the total organic acids [4]. Therefore, juices made from lulo are often perceived as being acidic, despite the use of sweeteners and the addition of water. The presence of the mesocarp, which is less acidic than the juice, can explain the lower perception of acidity in the juices to which it was added. Regarding the aroma, the role of pulp in the sensory perception of flavor in orange juice has been studied previously [50,51]. Pulp strongly influenced odor, aroma, and flavor perceptions [51]. Hydrophobic aroma compounds have been observed to interact with insoluble solids; consequently, the release of aroma compounds was higher in juice with lower pulp content [51]. The lower percentage of fruit in the pulped samples tested in our research may also explain the lower perception of fruit characteristic aromas.

Regarding consumer’s acceptability, the average hedonic score for samples ranged from 4.9 (2.0) to 6.0 (1.7). Thus, although no product was rated negatively, there is room for improvement in terms of the product’s sensory characteristics. Products containing only stevia as the sweetener were scored lower than products elaborated with the mixed sweeteners. One of the major problems with the use of stevia is its bitter aftertaste, which is detrimental to the acceptance of products containing this sweetener. Research [52] evaluating sweeteners (stevia, erythritol, and xylitol), using temporal-check-all-that-apply (TCATA) analysis, found that stevia had a much higher area under the curve (AUC) than the other sweeteners for undesirable attributes, such as bitter, metallic, and chemical flavors. However, previous studies have shown that by using a stevia with a higher percentage (97%) of rebaudioside A, the characteristic is much more similar to that of sucrose [53]. Other studies [54] have described how the use of sweetener blends provides flavors closer to a sucrose flavor, masking undesirable tastes (bitterness and metallic). This may explain the better scores obtained by products made with the sweetener mix compared to those made only with stevia.

Although the largest differences between the samples were observed in color, the overall acceptance (liking/disliking) scores do not show statistically significant differences (*p* < 0.05) between Js samples (more frequently described as green) and Ps samples (more frequently described as yellow). However, in South America, dark green lulo pulp obtained from the hybrid “La Selva” is highly valued by the industry due to its high acceptability [55]. In other beverages reconstituted from grapefruit powder, acceptability has been positively correlated with attributes such as pleasant taste, grapefruit aroma, and grapefruit taste, and it correlates negatively with bitter, not overly sweet, and artificial taste [49].

The penalty analysis enabled the identification of drivers for product improvement to increase the global acceptance for consumers. The cloudy appearance, off-odor, and very acidic flavor for the samples with juice only, and the cloudy appearance and chemical flavor for the pulped products, were the characteristics that most negatively impacted whether or not subjects liked the samples. Chemical flavor can be clearly related with stevia [52]. The JIS sample stood out for being cloudy and opaque. Although no studies have been found that directly relate the presence of stevia and inulin to turbidity and cloudiness in beverages, studies on mango nectar [56] showed that inulin and stevia may have a synergistic effect on some physical characteristics, such as viscosity.

## 5. Conclusions

The results of this research showed that differences were perceived between Js and Ps samples; Js samples were more often described as greener, while Ps samples were mostly described as yellow. Regarding flavor characteristics, Js samples were more often described as acidic compared to Ps samples. Samples with only stevia as a sweetener had negative flavor characteristics.

The soluble lulo beverage was accepted by consumers, but the use of stevia alone as a sweetener decreased the acceptability.

The characteristics of the products evaluated were far from the ideal product, but the herein presented research identified drivers of liking useful to improve the product’s sensory characteristics to increase consumer’s acceptance. For the Js samples, to increase acceptance, the attributes “bright”, “clean”, “ripe fruit”, “fruity” and “citrus” odor, “just-right acidity”,” just-right sweetness”, “sweet flavor”, and “fresh” flavor were “must haves” in the lulo-powder-based beverage. For Ps samples, the attributes “sweet”, “tropical fruit”, “citrus” and “pineapple” odor, “just-right sweetness”, just-right acidity”, and “fresh” flavor were necessary. The attributes “off-odor”, “very acidic” flavor, and “cloudy” in Js samples, and the attributes “chemical/artificial” flavor and “cloudy” in the Ps samples, were the characteristics that most negatively impacted on the hedonic evaluation of the products.

During the current investigation, the pandemic situation made the participation of a larger number of consumers difficult. However, the findings in this work provide a knowledge base for future studies with a larger number of participants, allowing consumer segments to be considered. The characterization of the products and drivers of liking found in this work can be used to improve reformulation of these products for future studies.

## Figures and Tables

**Table 1:** Formulation of 100 g of lulo beverage before dehydration.

Sample *	Juice (g)	Pulp (g)	Citric Acid (g)	Ascorbic Acid (g)	Maltodextrin (g)	Inulin (g)	Erythritol (g)	Xylitol (g)	Stevia (g)	Water (g)
JMS	48.8	0	0	0	4.9	0	0	0	0.029	46.4
JIS	48.8	0	0	0	0	4.9	0	0	0.029	46.4
JMX	45.7	0	0	0	4.6	0	5.0	1.3	0.016	43.5
JIX	45.7	0	0	0	0	4.6	5.0	1.3	0.016	43.5
PMS	0	32.0	0.10	0.48	3.2	0	0	0	0.029	64.1
PIS	0	32.0	0.10	0.48	0	3.2	0	0	0.029	64.1
PMX	0	30.0	0.09	0.45	3.0	0	5.0	1.3	0.016	60.0
PIX	0	30.0	0.09	0.45	0	3.0	5.0	1.3	0.016	60.0

* J = juice, P = pulp, M = maltodextrin, I = inulin, S = stevia, and X = erythritol + xylitol + stevia.

**Table 2:** Visual, olfactory, and gustatory attributes included in the CATA questions and their abbreviations.

Visual Attributes		Odor-Related Attributes		Flavor-Related Attributes	
Term	Abbreviation	Term	Abbreviation	Term	Abbreviation
Cloudy	V–Cloudy	Very aromatic	O–Aromatic	Sweet	F–Sweet
Clean	V–Clean	Not very aromatic	O–N.Aromatic	Very acidic	F–V.Acidic
Opaque	V–Opaque	Fruity	O–Fruity	Not very acidic	F–N.Acidic
Green color	V–Green	Floral	O–Floral	Very sweet	F–V.Sweet
Yellow color	V–Yellow	Chemical/artificial	O–Chemical	Not very sweet	F–N.Sweet
Brown color	V–Brown	Unripe fruit	O–Unripe	Just-right acidity	F–J.Acidity
Homogeneous appearance	V–Homogeneous	Citrus	O–Citrus	Just-right sweetness	F–J.Sweetness
Heterogeneous appearance	V–Heterogeneous	Tropical fruit	O–T.Fruit	Bitter	F–Bitter
Sediments	V–Sediments	Moldy/musty	O–Moldy	Metallic	F–Metallic
Very intense color	V–V.Color	Ripe fruit	O–Ripe	Fresh	F–Fresh
Not very intense color	V–N.Color	Sweet	O–Sweet	Off-flavor	F–Off
Bright	V–Bright	Medicine	O–Medicine	Unripe fruit	F–Unripe
		Off-odor	O–Off	Ripe fruit	F–Ripe
		Kiwi aroma	O–Kiwi	Rough	F–Rough
		Pineapple aroma	O–Pineapple	Chemical/artificial	F–Chemical
		Lemon aroma	O–Lemon	Dryness	F–Dryness
		Orange aroma	O–Orange	Liquorous	F–Liquorous
				Kiwi flavor	F–Kiwi
				Pineapple flavor	F–Pineapple
				Lemon flavor	F–Lemon
				Orange flavor	F–Orange

**Table 3:** Frequency of selection (%) for attributes for which a statistically significant difference (*p* < 0.05) among samples was found by using Cochran’s Q test.

Attributes	Ideal	Samples *							
		JMS	JIS	JMX	JIX	PMS	PIS	PMX	PIX
Visual (V)									
V-V.Color	56.5	5.8 ^c^	7.2 ^bc^	15.9 ^abc^	8.7 ^bc^	24.6 ^ab^	33.3 ^a^	30.4 ^a^	23.2 ^abc^
V-Clean	53.6	15.9 ^ab^	1.4 ^b^	17.4 ^a^	13.0 ^ab^	18.8 ^a^	8.7 ^ab^	15.9 ^ab^	13.0 ^ab^
V-Bright	44.9	10.1 ^ab^	0.0 ^b^	14.5 ^ab^	11.6 ^ab^	14.5 ^ab^	13.0 ^ab^	11.6 ^ab^	17.4 ^a^
V-Yellow	43.5	21.7 ^b^	11.6 ^b^	24.6 ^b^	23.2 ^b^	65.2 ^a^	63.8 ^a^	66.7 ^a^	68.1 ^a^
V-Green	13.0	65.2 ^a^	68.1 ^a^	73.9 ^a^	63.8 ^a^	2.9 ^b^	2.9 ^b^	1.4 ^b^	1.4 ^b^
V-Opaque	7.2	26.1 ^b^	50.7 ^a^	23.2 ^b^	26.1 ^b^	24.6 ^b^	39.1 ^ab^	31.9 ^ab^	27.5 ^b^
V-Sediments	7.2	29.0 ^bcd^	14.5 ^d^	23.2 ^cd^	34.8 ^abc^	42.0 ^abc^	49.3 ^a^	46.4 ^ab^	42.0 ^abc^
V-Cloudy	4.3	37.7 ^b^	63.8 ^a^	37.7 ^b^	40.6 ^b^	30.4 ^b^	27.5 ^b^	30.4 ^b^	31.9 ^b^
V-N.Color	4.3	27.5 ^a^	26.1 ^ab^	21.7 ^ab^	23.2 ^ab^	8.7 ^b^	15.9 ^ab^	10.1 ^ab^	18.8 ^ab^
V-Heterogeneous	1.4	13.0 ^b^	15.9 ^ab^	10.1 ^b^	15.9 ^ab^	11.6 ^b^	13.0 ^b^	20.3 ^ab^	29.0 ^a^
Odor (O)									
O-Aromatic	43.5	15.9 ^ab^	21.7 ^a^	18.8 ^ab^	18.8 ^ab^	11.6 ^ab^	8.7 ^ab^	13.0 ^ab^	5.8 ^b^
O-Ripe	26.1	10.1 ^ab^	14.5 ^ab^	7.2 ^b^	11.6 ^ab^	15.9 ^ab^	23.2 ^a^	17.4 ^ab^	13.0 ^ab^
O-N.Aromatic	4.3	20.3 ^b^	29.0 ^ab^	24.6 ^b^	18.8 ^b^	34.8 ^ab^	39.1 ^ab^	36.2 ^ab^	49.3 ^a^
Flavor (F)									
F-J.Acidity	66.7	33.3 ^ab^	26.1 ^ab^	33.3 ^ab^	46.4 ^a^	20.3 ^b^	24.6 ^ab^	31.9 ^ab^	40.6 ^ab^
F-Sweet	31.9	15.9 ^ab^	13.0 ^ab^	15.9 ^ab^	8.7 ^b^	17.4 ^ab^	20.3 ^ab^	20.3 ^ab^	27.5 ^a^
F-V.Acidic	7.2	31.9 ^ab^	40.6 ^a^	36.2 ^a^	31.9 ^ab^	15.9 ^bc^	11.6 ^c^	10.1 ^c^	11.6 ^c^
F-Chemical	0.0	18.8 ^abc^	21.7 ^abc^	7.2 ^c^	13.0 ^bc^	33.3 ^a^	29.0 ^ab^	17.4 ^abc^	10.1 ^c^

Distinct superscripts letters (^a,b,c,d^) within a row indicate that products differ significantly (*p* < 0.05) according to the multiple-pairwise-comparisons Sheskin critical-difference procedure. * P = pulp, J = juice, M = maltodextrin, I = inulin, S = stevia, and X = erythritol + xylitol + stevia. Ideal = ideal product.

**Table 4:** Frequency of selection (%) for attributes for which a statistically significant difference (*p* < 0.05) among samples was found by using Cochran’s Q test for each type of sample (with juice only or with pulp).

Attributes	Ideal	Juice Only Samples *			
		JMS	JIS	JMX	JIX
Visual (V)					
V-Clean	53.6	15.9 ^a^	1.4 ^b^	17.4 ^a^	13.0 ^ab^
V-Bright	44.9	10.1 ^ab^	0.0 ^b^	14.5 ^a^	11.6 ^a^
V-Opaque	7.2	26.1 ^b^	50.7 ^a^	23.2 ^b^	26.1 ^b^
V-Sediments	7.2	29.0 ^ab^	14.5 ^b^	23.2 ^ab^	34.8 ^a^
V-Cloudy	4.3	37.7 ^b^	63.8 ^a^	37.7 ^b^	40.6 ^b^
Flavor-related (F)					
F-J.Acidity	66.7	33.3 ^ab^	26.1 ^b^	33.3 ^ab^	46.4 ^a^
F-Bitter	1.4	23.2 ^a^	17.4 ^ab^	7.2 ^b^	10.1 ^ab^
F-Metallic	0.0	15.9 ^a^	7.2 ^ab^	5.9 ^ab^	4.3 ^b^
F-Chemical	0.0	18.8 ^ab^	21.7 ^a^	7.2 ^b^	13.0 ^ab^
	**Ideal**	With pulp samples *			
		**PMS**	**PIS**	**PMX**	**PIX**
Visual (V)					
V-Homogeneous	72.5	40.6 ^a^	30.4 ^ab^	33.3 ^ab^	26.1 ^b^
V-Heterogeneous	1.4	11.6 ^b^	13.0 ^b^	20.3 ^ab^	29.0 ^a^
Odor-related (O)					
O-Citrus	50.7	15.9 ^ab^	27.5 ^a^	13.0 ^b^	23.2 ^ab^
O-Medicine	0.0	18.8 ^a^	14.5 ^ab^	10.1 ^ab^	7.2 ^b^
Flavor-related (F)					
F-J.Acidity	66.7	20.3 ^b^	24.6 ^ab^	31.9 ^ab^	40.6 ^a^
F-Off	0.0	26.1 ^a^	27.5 ^a^	11.6 ^b^	14.5 ^ab^
F-Chemical	0.0	33.3 ^a^	29.0 ^a^	17.4 ^ab^	10.1 ^b^

Distinct superscripts letters (^a,b^) in a row indicate that products differ significantly (*p* < 0.05) according to the multiple pairwise comparisons Sheskin critical difference procedure. * P = pulp, J = juice, M = maltodextrin, I = inulin, S = stevia, and X = erythritol + xylitol + stevia. Ideal = ideal product.

**Table 5:** Hedonic scores (mean and standard deviation) of the lulo (*Solanum quitoense* Lam.) beverage evaluated by consumers (*n* = 69).

Sample *	Hedonic Score ^1^
PMX	6.0 (1.7) ^a^
JMX	5.9 (1.8) ^ab^
PIX	5.9 (1.7) ^ab^
JIX	5.9 (1.9) ^ab^
PIS	5.3 (2.0) ^bc^
JMS	5.0 (1.9) ^c^
JIS	5.0 (2.0) ^c^
PMS	4.9 (2.0) ^c^

^1^ Distinct superscripts letters (^a,b,c^) indicate that products differ significantly (*p* < 0.05) by the Fisher’s least-significant-differences (LSD) test. * P = pulp, J = juice, M = maltodextrin, I = inulin, S = stevia, and X = erythritol + xylitol + stevia.

## Data Availability

Data is contained within the article or Supplementary Material.

## Supplementary Materials

The following supporting information can be downloaded at https://www.mdpi.com/article/10.3390/foods11193129/s1. Table S1: Frequency (%) of the visual terms related of the CATA questions used by consumers to describe the eight beverage samples and their ideal product. Table S2: Frequency (%) of the odor terms related to the CATA questions used by consumers to describe the eight beverage samples and their ideal product. Table S3: Frequency (%) of the flavor terms related of the CATA question used by consumers to describe the eight beverage samples and their ideal product. Figure S1: Principal component analysis (PCA) applied to the correlation coefficients between attributes and liking scores. Table S4. Penalty analysis for juice only samples (Js). Summary of the frequencies with which presence in the ideal but not in the real product (P(No)|(Yes)) occurs and presence in the ideal and real product (P(Yes)|(Yes)) occurs for each attribute, mean drops in liking between the two situations, and significances. Table S5. Penalty analysis for juice-only samples (Js). Summary of the frequencies with which no presence in the ideal nor in the real product (P(No)|(No)) occurs and no presence in the ideal but yes in the real product (P(Yes)|(No)) occurs for each attribute, mean drops in liking between the two situations, and significances. Table S6. Penalty analysis for samples with pulp (Ps). Summary of the frequencies with which presence in the ideal but not in the real product (P(No)|(Yes)) occurs and presence in the ideal and real product (P(Yes)|(Yes)) occurs for each attribute, mean drops in liking between the two situations, and significances. Table S7. Penalty analysis for samples with pulp (Ps). Summary of the frequencies with which no presence in the ideal nor in the real product (P(No)|(No)) occurs and no presence in the ideal but yes in the real product (P(Yes)|(No)) occurs for each attribute, mean drops in liking between the two situations, and significances.
