# Supplementary material for: Sensory Characterization and Acceptability of a New Lulo (Solanum quitoense Lam.) Powder-Based Soluble Beverage Using Rapid Evaluation Techniques with Consumers
Source: Foods. 2022 Oct 8;11(19):3129. doi: 10.3390/foods11193129 (PMC9564273; doi:10.3390/foods11193129)
Supplement: Supplementary file 1 [file foods-11-03129-s001.zip › foods-1889901-supplementary.pdf]

foods MDPI

Table S1. Frequency (%) with the visual terms related of the CATA questions used by consumers to describe the eight beverage samples and their ideal product.

Atributtes	Ideal	JMS	JIS	JMX	JIX	PMS	PIS	PMX	PIX
V-Homogeneous	72.5	29.0	39.1	36.2	27.5	39.1	30.4	33.3	26.1
V-V.Color	56.5	5.8	7.2	15.9	8.7	23.2	33.3	30.4	23.2
V-Clean	53.6	15.9	1.4	17.4	13.0	18.8	8.7	15.9	13.0
V-Bright	44.9	10.1	0.0	14.5	11.6	13.0	13.0	11.6	17.4
V-Yellow	43.5	21.7	11.6	24.6	23.2	63.8	63.8	66.7	68.1
V-Green	13.0	65.2	68.1	73.9	63.8	2.9	2.9	1.4	1.4
V-Opaque	7.2	26.1	50.7	23.2	26.1	23.2	39.1	31.9	27.5
V-Brown	7.2	20.3	23.2	20.3	21.7	11.6	10.1	10.1	7.2
V-Sediments	7.2	29.0	14.5	23.2	34.8	42.0	49.3	46.4	42.0
V-Cloudy	4.3	37.7	63.8	37.7	40.6	29.0	27.5	30.4	31.9
V-N.Color	4.3	27.5	26.1	21.7	23.2	8.7	15.9	10.1	18.8
V-Heterogeneous	1.4	13.0	15.9	10.1	15.9	11.6	13.0	20.3	29.0

Table S2. Frequency (%) with the odor terms related of the CATA questions used by consumers to describe the eight beverage samples and their ideal product.

Atributtes	Ideal	JMS	JIS	JMX	JIX	PMS	PIS	PMX	PIX
O-T.Fruit	63.8	33.3	34.8	43.5	40.6	36.2	36.2	46.4	36.2
O-Fruity	53.6	33.3	29.0	39.1	31.9	33.3	30.4	30.4	40.6
O-Citrus	50.7	29.0	31.9	24.6	31.9	15.9	27.5	13.0	23.2
O-Aromatic	43.5	15.9	21.7	18.8	18.8	10.1	8.7	13.0	5.8
O-Sweet	33.3	7.2	5.8	11.6	13.0	13.0	10.1	14.5	13.0
O-Ripe	26.1	10.1	14.5	7.2	11.6	14.5	23.2	17.4	13.0
O-Pineapple	18.8	20.3	17.4	18.8	17.4	13.0	17.4	15.9	17.4
O-Orange	17.4	1.4	2.9	0.0	0.0	4.3	7.2	4.3	4.3
O-Floral	14.5	13.0	8.7	10.1	11.6	7.2	10.1	7.2	8.7
O-Kiwi	8.7	8.7	13.0	14.5	17.4	8.7	4.3	5.8	7.2
O-Unripe	5.8	20.3	20.3	20.3	20.3	13.0	18.8	11.6	14.5
O-Lemon	5.8	5.8	5.8	5.8	2.9	1.4	0.0	1.4	5.8
O-N.Aromatic	4.3	20.3	29.0	24.6	18.8	34.8	39.1	36.2	49.3
O-Chemical	0.0	10.1	18.8	10.1	14.5	15.9	10.1	10.1	7.2
O-Moldy	0.0	5.8	5.8	2.9	4.3	4.3	1.4	2.9	2.9
O-Medicine	0.0	13.0	11.6	5.8	13.0	18.8	14.5	10.1	7.2
O-Off	0.0	24.6	18.8	15.9	21.7	23.2	17.4	13.0	13.0

Table S3. Frequency (%) with the flavor terms related of the CATA questions used by consumers to describe the eight beverage samples and their ideal product.

Atributtes	Ideal	JMS	JIS	JMX	JIX	PMS	PIS	PMX	PIX
F-J.Acidity	66.7	33.3	26.1	33.3	46.4	20.3	24.6	31.9	40.6
F-Fresh	56.5	17.4	8.7	15.9	23.2	7.2	10.1	14.5	18.8
F-J.Sweetness	53.6	11.6	14.5	26.1	27.5	15.9	15.9	23.2	26.1
F-Sweet	31.9	15.9	13.0	15.9	8.7	17.4	20.3	20.3	27.5
F-Ripe	31.9	8.7	10.1	7.2	2.9	15.9	15.9	15.9	15.9
F-Pineapple	31.9	17.4	20.3	27.5	18.8	13.0	20.3	24.6	17.4
F-Orange	24.6	2.9	8.7	11.6	11.6	7.2	8.7	8.7	14.5
F-Kiwi	15.9	8.7	14.5	15.9	17.4	4.3	4.3	13.0	11.6
F-N.Sweet	14.5	30.4	18.8	17.4	21.7	23.2	18.8	15.9	14.5
F-Lemon	14.5	13.0	11.6	14.5	14.5	4.3	5.8	5.8	10.1
F-N.Acidic	11.6	14.5	11.6	11.6	8.7	24.6	24.6	21.7	18.8
F-V.Acidic	7.2	31.9	40.6	36.2	31.9	14.5	11.6	10.1	11.6
F-Unripe	7.2	18.8	18.8	17.4	21.7	15.9	10.1	10.1	8.7
F-V.Sweet	4.3	7.2	7.2	1.4	10.1	5.8	8.7	4.3	8.7
F-Rough	2.9	18.8	18.8	20.3	13.0	13.0	14.5	13.0	11.6
F-Bitter	1.4	23.2	17.4	7.2	10.1	15.9	15.9	13.0	14.5
F-Metallic	0.0	15.9	7.2	5.8	4.3	11.6	13.0	7.2	2.9
F-Off	0.0	23.2	26.1	13.0	14.5	24.6	27.5	11.6	14.5
F-Chemical	0.0	18.8	21.7	7.2	13.0	31.9	29.0	17.4	10.1
F-Dryness	0.0	10.1	11.6	10.1	4.3	15.9	11.6	10.1	7.2
F-Liquorous	0.0	1.4	0.0	0.0	0.0	2.9	4.3	1.4	4.3

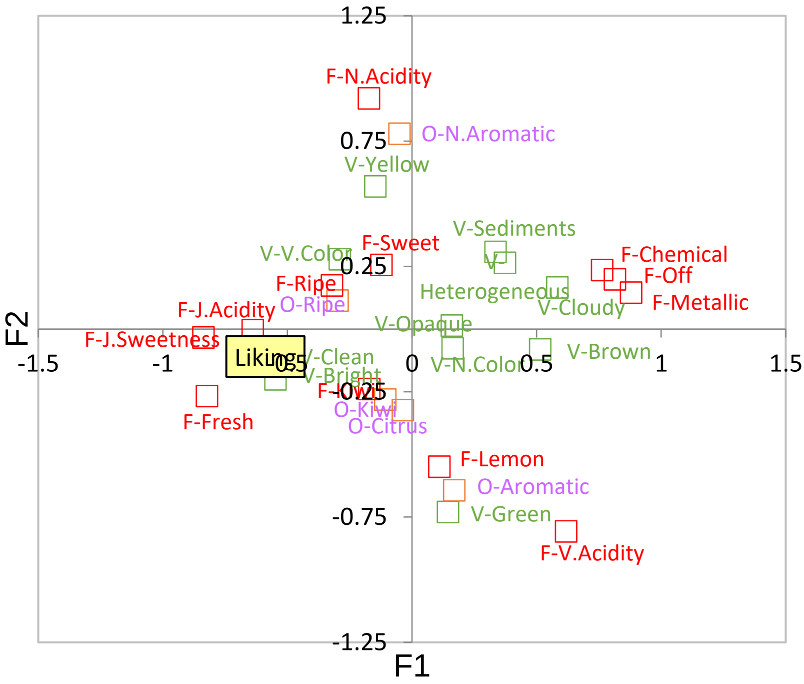

Figure S1. Principal component analysis (PCA) applied to the correlation coefficients between attributes and liking scores.

Table S4. Penalty analysis for juice only samples (Js). Summary of the frequencies with which presence in the ideal but not in the real product (P(No)|(Yes)) and presence in the ideal and real product (P(Yes)|(Yes)) occurs for each attribute, mean drops in liking between the two situations and significances.

Variable	Level	Frequency %	Mean (Liking)	Effect on the mean	P-Value	Significant	Penalty
Clean	P(No)I(Yes)	43.48%	5.008	1.170	0.011	Yes	
	P(Yes)I(Yes)	10.14%	6.179				0.808
Yellow color	P(No)I(Yes)	34.78%	5.302	0.073	0.985	No	
	P(Yes)I(Yes)	8.70%	5.375				-0.085
Homogeneous appearance	P(No)I(Yes)	46.01%	5.118	0.731	0.028	Yes	
	P(Yes)I(Yes)	26.45%	5.849				0.539
Heterogeneous appearance	P(No)I(Yes)	1.45%	5.750		<0.0001	No	0.000
	P(Yes)I(Yes)	0.00%			<0.0001	No	0.000
Very intense color	P(No)I(Yes)	49.28%	5.199	0.901	0.129	No	
	P(Yes)I(Yes)	7.25%	6.100				0.698
Bright	P(No)I(Yes)	39.49%	4.633	2.567	<0.0001	Yes	
	P(Yes)I(Yes)	5.43%	7.200				1.848
Very aromatic	P(No)I(Yes)	30.80%	6.012	-0.383	0.580	No	
	P(Yes)I(Yes)	12.68%	5.629				0.201
Fruity	P(No)I(Yes)	30.43%	4.869	1.256	0.000	Yes	
	P(Yes)I(Yes)	23.19%	6.125				0.875
Chemical/artificial	P(No)I(Yes)	0.00%			<0.0001	No	0.000
	P(Yes)I(Yes)	0.00%			<0.0001	No	0.000
Citrus	P(No)I(Yes)	29.71%	4.890	0.817	0.037	Yes	
	P(Yes)I(Yes)	21.01%	5.707				0.322
Tropical fruit	P(No)I(Yes)	32.97%	5.341	0.389	0.385	No	
	P(Yes)I(Yes)	30.80%	5.729				0.400
Moldy/musty	P(No)I(Yes)	0.00%			<0.0001	No	0.000
	P(Yes)I(Yes)	0.00%			<0.0001	No	0.000
Ripe fruit odor	P(No)I(Yes)	20.65%	4.912	1.754	0.005	Yes	
	P(Yes)I(Yes)	5.43%	6.667				1.284
Sweet odor	P(No)I(Yes)	28.62%	4.949	1.204	0.095	No	
	P(Yes)I(Yes)	4.71%	6.154				0.736
Medicine	P(No)I(Yes)	0.00%			<0.0001	No	0.000
	P(Yes)I(Yes)	0.00%			<0.0001	No	0.000
Off-odor	P(No)I(Yes)	0.00%			<0.0001	No	0.000
	P(Yes)I(Yes)	0.00%			<0.0001	No	0.000
Orange aroma	P(No)I(Yes)	17.39%	4.917		<0.0001	No	0.000
	P(Yes)I(Yes)	0.00%			<0.0001	No	0.000
Sweet flavor	P(No)I(Yes)	24.28%	4.522	1.192	0.032	Yes	
	P(Yes)I(Yes)	7.61%	5.714				0.283
Not very acidic	P(No)I(Yes)	11.59%	4.344		<0.0001	No	0.000
	P(Yes)I(Yes)	0.00%			<0.0001	No	0.000
Just-right acidity	P(No)I(Yes)	39.13%	5.074	1.347	<0.0001	Yes	

	P(Yes)I(Yes)	27.54%	6.421				1.336
Just-right sweetness	P(No)I(Yes)	40.22%	5.099	2.252	<0.0001	Yes	
	P(Yes)I(Yes)	13.41%	7.351				2.192
Metallic	P(No)I(Yes)	0.00%			<0.0001	No	0.000
	P(Yes)I(Yes)	0.00%			<0.0001	No	0.000
Fresh	P(No)I(Yes)	44.57%	5.033	1.816	<0.0001	Yes	
	P(Yes)I(Yes)	11.96%	6.848				1.585
Off-flavor	P(No)I(Yes)	0.00%			<0.0001	No	0.000
	P(Yes)I(Yes)	0.00%			<0.0001	No	0.000
Ripe fruit flavor	P(No)I(Yes)	30.43%	5.071	2.179	0.073	No	
	P(Yes)I(Yes)	1.45%	7.250				1.824
Chemical/artificial	P(No)I(Yes)	0.00%			<0.0001	No	0.000
	P(Yes)I(Yes)	0.00%			<0.0001	No	0.000
Dryness	P(No)I(Yes)	0.00%			<0.0001	No	0.000
	P(Yes)I(Yes)	0.00%			<0.0001	No	0.000
Liquorous	P(No)I(Yes)	0.00%			<0.0001	No	0.000
	P(Yes)I(Yes)	0.00%			<0.0001	No	0.000

Table S5. Penalty analysis for juice only samples (Js). Summary of the frequencies with which no presence in the ideal nor in the real product (P(No)|(No)) and no presence in the ideal but yes in the real product (P(Yes)|(No)) occurs for each attribute, mean drops in liking between the two situations and significances.

Variable	Level	Frequency %	Mean (Liking)	Effects on the mean	P-Value	Significant	Penalty
Cloudy	P(No)I(No)	54.35%	5.847				0.863
	P(Yes)I(No)	41.30%	4.921	-0.926	0.000	Yes	
Opaque	P(No)I(No)	64.86%	5.559				0.301
	P(Yes)I(No)	27.90%	5.091	-0.468	0.075	No	
Green color	P(No)I(No)	30.07%	5.157				-0.424
	P(Yes)I(No)	56.88%	5.338	0.181	0.480	No	
Sediments	P(No)I(No)	70.65%	5.508				0.187
	P(Yes)I(No)	22.10%	5.148	-0.360	0.216	No	
Not very intense color	P(No)I(No)	72.83%	5.498				0.164
	P(Yes)I(No)	22.83%	5.222	-0.275	0.320	No	
Not very aromatic	P(No)I(No)	73.19%	5.530				0.286
	P(Yes)I(No)	22.46%	5.371	-0.159	0.580	No	
Off-odor	P(No)I(No)	79.71%	5.755				
	P(Yes)I(No)	20.29%	4.268	-1.487	<0.0001	Yes	0.000
Very acidic	P(No)I(No)	61.96%	5.702				0.654
	P(Yes)I(No)	30.80%	4.565	-1.137	<0.0001	Yes	

Table S6. Penalty analysis for samples with pulp (Ps). Summary of the frequencies with which presence in the ideal but not in the real product (P(No)|(Yes)) and presence in the ideal and real product (P(Yes)|(Yes)) occurs for each attribute, mean drops in liking between the two situations and significances.

Variable	Level	Frequency %	Mean (Liking)	Effect on the mean	P-Value	Significant	Penalty
Clean	P(No)I(Yes)	42.39%	5.436	0.790	0.102	No	
	P(Yes)I(Yes)	11.23%	6.226				0.818
Green color	P(No)I(Yes)	13.04%	5.500		<0.0001	No	0.000
	P(Yes)I(Yes)	0.00%			<0.0001	No	0.000
Homogeneous appearance	P(No)I(Yes)	46.01%	5.315	0.534	0.140	No	
	P(Yes)I(Yes)	26.45%	5.849				0.475
Heterogeneous appearance	P(No)I(Yes)	1.45%	4.750		<0.0001	No	0.000
	P(Yes)I(Yes)	0.00%			<0.0001	No	0.000
Very intense color	P(No)I(Yes)	38.04%	5.029	0.579	0.172	No	
	P(Yes)I(Yes)	18.48%	5.608				0.132
Bright	P(No)I(Yes)	35.51%	5.122	-0.584	0.332	No	
	P(Yes)I(Yes)	9.42%	4.538				-1.062
Very aromatic	P(No)I(Yes)	35.51%	5.316	-0.180	0.916	No	
	P(Yes)I(Yes)	7.97%	5.136				-0.395
Fruity	P(No)I(Yes)	28.99%	5.288	0.330	0.551	No	
	P(Yes)I(Yes)	24.64%	5.618				0.156
Chemical/artificial odor	P(No)I(Yes)	0.00%			<0.0001	No	0.000
	P(Yes)I(Yes)	0.00%			<0.0001	No	0.000
Citrus	P(No)I(Yes)	35.51%	4.959	1.112	0.004	Yes	
	P(Yes)I(Yes)	15.22%	6.071				0.674
Tropical fruit	P(No)I(Yes)	31.52%	4.897	1.126	0.000	Yes	
	P(Yes)I(Yes)	32.25%	6.022				0.771
Moldy/musty	P(No)I(Yes)	0.00%			<0.0001	No	0.000
	P(Yes)I(Yes)	0.00%			<0.0001	No	0.000
Sweet odor	P(No)I(Yes)	27.54%	5.132	1.431	0.018	Yes	
	P(Yes)I(Yes)	5.80%	6.563				1.128
Medicine	P(No)I(Yes)	0.00%			<0.0001	No	0.000
	P(Yes)I(Yes)	0.00%			<0.0001	No	0.000
Off-odor	P(No)I(Yes)	0.00%			<0.0001	No	0.000
	P(Yes)I(Yes)	0.00%			<0.0001	No	0.000
Lemon aroma	P(No)I(Yes)	5.80%	4.938		<0.0001	No	0.000
	P(Yes)I(Yes)	0.00%			<0.0001	No	0.000
Sweet flavor	P(No)I(Yes)	22.10%	5.066	0.860	0.127	No	
	P(Yes)I(Yes)	9.78%	5.926				0.472
Just-right acidity	P(No)I(Yes)	45.29%	4.920	1.521	<0.0001	Yes	
	P(Yes)I(Yes)	21.38%	6.441				1.196
Just-right sweetness	P(No)I(Yes)	36.96%	5.480	1.367	<0.0001	Yes	
	P(Yes)I(Yes)	16.67%	6.848				1.617

Metallic	P(No)I(Yes)	0.00%			<0.0001	No	0.000
	P(Yes)I(Yes)	0.00%			<0.0001	No	0.000
Fresh	P(No)I(Yes)	46.38%	5.063	1.795	<0.0001	Yes	
	P(Yes)I(Yes)	10.14%	6.857				1.510
Off-flavor	P(No)I(Yes)	0.00%			<0.0001	No	0.000
	P(Yes)I(Yes)	0.00%			<0.0001	No	0.000
Ripe fruit flavor	P(No)I(Yes)	27.17%	5.587	1.259	0.072	No	
	P(Yes)I(Yes)	4.71%	6.846				1.413
Chemical/artificial flavor	P(No)I(Yes)	0.00%			<0.0001	No	0.000
	P(Yes)I(Yes)	0.00%			<0.0001	No	0.000
Dryness	P(No)I(Yes)	0.00%			<0.0001	No	0.000
	P(Yes)I(Yes)	0.00%			<0.0001	No	0.000
Liquorous	P(No)I(Yes)	0.00%			<0.0001	No	0.000
	P(Yes)I(Yes)	0.00%			<0.0001	No	0.000
Pineapple flavor	P(No)I(Yes)	22.83%	5.254	1.266	0.014	Yes	
	P(Yes)I(Yes)	9.06%	6.520				1.122

Table S7. Penalty analysis for samples with pulp (Ps). Summary of the frequencies with which no presence in the ideal nor in the real product (P(No)|(No)) and no presence in the ideal but yes in the real product (P(Yes)|(No)) occurs for each attribute, mean drops in liking between the two situations and significances.

Variable	Level	Frequency %	Mean (Liking)	Effects on the mean	P-Value	Significant	Penalty
Cloudy	P(No)I(No)	69.57%	5.677				0.582
	P(Yes)I(No)	26.09%	5.125	-0.552	0.039	Yes	
Opaque	P(No)I(No)	65.58%	5.630				0.377
	P(Yes)I(No)	27.17%	5.413	-0.217	0.401	No	
Yellow color	P(No)I(No)	26.81%	5.419				-0.111
	P(Yes)I(No)	29.71%	4.976	-0.443	0.308	No	
Sediments	P(No)I(No)	53.26%	5.605				0.226
	P(Yes)I(No)	39.49%	5.514	-0.092	0.704	No	
Not very aromatic	P(No)I(No)	57.97%	5.619				0.283
	P(Yes)I(No)	37.68%	5.317	-0.301	0.219	No	
Chemical/artificial odor	P(No)I(No)	77.54%	6.009				
	P(Yes)I(No)	22.46%	3.742	-2.267	<0.0001	Yes	0.000
